# Comparative Analysis of Arsenic Transport and Tolerance Mechanisms: Evolution from Prokaryote to Higher Plants

**DOI:** 10.3390/cells11172741

**Published:** 2022-09-02

**Authors:** Jie Zhang, Jiayou Liu, Fubin Zheng, Min Yu, Sergey Shabala, Won-Yong Song

**Affiliations:** 1 International Centre for Environmental Membrane Biology, Department of Horticulture, School of Food Science and Engineering, Foshan University, Foshan 528011, China; 2 Tasmanian Institute of Agriculture, University of Tasmania, Hobart, 7001, Australia; 3 School of Biological Science, University of Western Australia, Perth 6009, Australia

**Keywords:** arsenic, arsenic operon, arsenate reductase, arsenite efflux, ATP Binding Cassette transporter

## Abstract

Arsenic (As) is a toxic metalloid for all living organisms and can cause serious harm to humans. Arsenic is also toxic to plants. To alleviate As toxicity, all living organisms (from prokaryotes to higher plants) have evolved comprehensive mechanisms to reduce cytosolic As concentration through the set of As transporters localized at the plasma and tonoplast membranes, which operate either in arsenite As(III) extrusion out of cells (via ArsB, ACR3, and aquaporins) or by sequestering arsenic into vacuoles (by ABC transporters). In addition, a special arsenate resistance mechanism found in some bacterial systems has evolved in an As hyperaccumulating fern *Pteris vittata*, which involves transforming arsenate As(V) to an As(V) phosphoglycerate derivative by a glyceraldehyde 3-phosphate dehydrogenase and transporting this complex by an efflux transporter. In the present review, we summarize the evolution of these arsenic resistance mechanisms from prokaryotes to eukaryotes and discuss future approaches that could be utilized to better understand and improve As resistance mechanisms in plants.

## 1. Introduction

Arsenic (As) is a toxic metalloid that possesses properties intermediate between metals and non-metals. Arsenic is highly toxic to humans, with low and non-cytotoxic concentrations of As greatly enhancing the carcinogenicity in mammalian systems [1]. According to the World Health Organization (WHO) and the U.S. Environmental Protection Agency (EPA), large areas of Bangladesh, India, China, Argentina, Ghana, Chile, Vietnam, Canada, Laos, Mexico, the United States, and several other countries are highly contaminated with As, exceeding the WHO threshold value of 10 μg/L [2]. Arsenic contamination of soils is also widely reported [2,3,4]. This contamination comes from both natural and anthropogenic sources. The latter includes herbicides, growth promoters for farm animals, wood preservatives, the semiconductor industry, mining, fossil fuel combustion, and other industrial sources [4,5]. The contamination with As has become a serious environmental and ecological problem and has led to serious toxicological issues, especially in the Southeast area of Asia, such as the West Bengal region of India and Bangladesh [4]. Arsenic can enter the human body either through consumption of As-contaminated drinking water or via the food chain. Arsenic contamination in drinking water affects more than 150 million people across the world [1]. More than 2/3 of these people live in South and Southeast Asia countries [1]. Foods such as rice and vegetables may also accumulate high quantities of As in their edible parts, posing significant health risks in humans [6]. 

Living organisms, from bacteria to higher plants, have evolved elaborate resistance mechanisms to deal with the toxic effects of As. In ancient times when arsenite (As(III)) was the major form of As, prokaryotic cells developed mechanisms to export As(III) by ArsB transporter [7,8]. After the rise of atmospheric oxygen during the Great Oxidation Event (GOE) ~2.4 billion years ago, arsenate (As(V)) gradually became the major form of As in the environment [9]. Since As(V) has high structural similarity with inorganic phosphate, prokaryotic cells take up As(V) by phosphate transporters, which is then quickly reduced to As(III) by two different types of ArsC proteins, thioredoxin-coupled and glutaredoxin-linked ArsC proteins [10]. In eukaryotes, the same process is mediated by ACR2 (Arsenics Compounds Resistance 2) or ARQ1 (Arabidopsis thaliana arsenate reductase QTL1)/HAC proteins (High Arsenic Content) [11,12,13]. ACR2 mediates As(V) reduction in yeast, while ARQs/HACs mainly work as arsenate reductases in plants. Then, As(III) is either exported out of the cells by either aquaglyceroporins (from aquaporins family) or As(III)/H^+^ antiporter (ACR3) or sequestered into vacuoles by ABC transporters [14]. Additionally, some other mechanisms for arsenic tolerance, such as arsenic methylation [5] and As(V) efflux [15,16], also exist. While the essentiality of these mechanisms is well established, it is not clear how they have evolved and what the functional and structural differences of the key proteins involved in the As resistance mechanisms between prokaryotes and eukaryotes are. While a comparative analysis of arsenic-tolerance mechanisms has been the subject of many reviews [8,10,17,18], this topic is rarely discussed in an evolutionary context. At the same time, a comparative analysis of the arsenic resistance mechanisms between prokaryotes and higher plants would be instrumental in a search for new mechanisms and new genes involved in arsenic resistance in higher plants, keeping in mind the need for reducing As accumulation in the edible crops.

## 2. Arsenic Reduction Mechanisms, from Prokaryote to Higher Plants

In the natural environment, Arsenic (As) exists mainly in the form of arsenite (As(III)) and arsenate(As(V)). Under anaerobic conditions, As mainly exists in a reduced form, As(III), and it is taken into living organisms via aquaporins or hexose transporters [6]. Under aerobic conditions, an oxidized form of arsenic, As(V), is abundant and can be transported through phosphate transporters. Once As(V) is taken up into cells, it will be rapidly reduced to As(III) by arsenate reductases, which will be further detoxified by either extrusion or vacuolar sequestration, followed by the conjugation of As(III) with thiols [6]. 

Arsenate reductases exist in almost all living organisms, including prokaryotes, yeast, and higher plants. The major pathways for arsenate reduction are mediated by arsenate reductases, including ArsC, ACR2, and HACs, which arose independently [19]. The mechanisms of arsenate reduction by these enzymes are different and independent, but the result is the same (arsenate reduction to arsenite by cysteine cascades) [19]. In addition to the above arsenate reductases, the arsenate respiring bacteria containing Arr operon consist of ArrA and ArrB, which could form a heterodimeric ArrAB arsenate reductase. Although the ArrAB respiratory arsenate reductase also transforms the pentavalent species, it is for energy production, not resistance [20].

**Figure 1:**
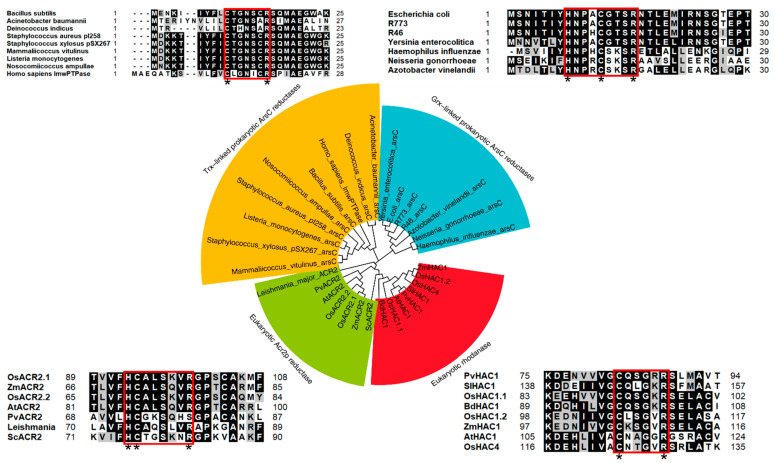
The phylogenetic tree of arsenate reductases in different organisms. Alignment of the amino acid sequences of arsenate reductases was made by Clustal W software using default parameters. The phylogenetic tree was made using MEGA X software by the Maximum Likelihood method and JTT matrix-based model. The bootstrap values (percentage) of 1000 replicates are shown at the branching points. The arsenate reductases can be divided into four subfamilies. Each subfamily contains different activation sites. The active sites of arsenate reductases are boxed in red. The * indicates conserved amino acid residues in the active sites. The accession numbers of the proteins are shown in Supplementary Table S1.

Prokaryote arsenate reductases can be divided into two different classes, thioredoxin (Trx)-linked and glutaredoxin (Grx)-linked ArsC classes (Figure 1). The Trx-linked ArsC is represented by the *Staphylococcus aureus* plasmid pI258–encoded ArsC gene product [21], which mediates arsenate reduction by several steps: (1) the nucleophilic attack of the thiol group of Cys10 on the arsenate substrate results in the formation of a covalent Cys10-S-AsHO_3_^−^ intermediate, (2) Cys82 attacks Cys10 with the formation of a Cys10-Cys82 disulfide intermediate, (3) Cys89 attacks Cys82, forming a Cys82-Cys89 disulfide, and (4) reduced ArsC is regenerated by thioredoxin (Trx) reduction of the oxidized Cys82-Cys89 disulfide [19]. The prokaryotic Grx-linked arsenate reductase includes ArsC protein from the *Escherichia coli* plasmid R773, which exhibits arsenate reductase activity dependent on the presence of reduced GSH and Grx without forming disulfide [22]. There is no relationship between the two types of ArsC proteins; they belong to different clades in the phylogenetic tree (Figure 1), and the Trx-linked and Grx-linked ArsC proteins contain different active sites, although the active sites of both types of ArsC proteins are localized at N-terminal (Figure 1). The active site of Trx-linked ArsC is CX_5_R, while the active site of Grx-linked ArsC is HX_3_CX_3_R [10,19]. The protein sequence and the structure of Trx-linked ArsC proteins have high similarity to low molecular weight protein phosphotyrosine phosphatases (lmwPTPase) [23]. However, the protein structure of R773 ArsC (Grx-linked type) is not related to other proteins [19]. Thus, the two families of prokaryote ArsC arsenate reductases may evolve separately. 

Eukaryotes, including yeasts and plants, do not have homologs of prokaryotes arsenate reductase. The eukaryotic arsenate reductases can be divided into two major groups, ACR2p-like proteins and rhodanese-like proteins (Figure 1). The first known arsenate reductase in eukaryotes is ACR2p from *Saccharomyces cerevisiae* [24,25]. ACR2p has independently evolved from the two prokaryote *arsC* classes, and it is a homolog of the Cdc25a cell-cycle protein tyrosine phosphatase (PTPase) [26]. Similar to the bacterial R773 ArsC, ACR2p also uses GSH and Grx as a source of reducing equivalents. However, ACR2p has a similar consensus active site of CX_5_R with the bacterial Trx-linked ArsC family [19,26]. However, the location of the catalytic cysteine residue of ACR2p is different from bacterial Trx-linked ArsC. The catalytic cysteine of the bacterial arsenate reductases is at the N-terminal region, but that of ACR2p is in the middle [19]. Although there are homologs of ACR2p in plants, their roles as arsenate reductase are not clearly identified yet. *Arabidopsis thaliana* ACR2 has an arsenate reductase activity in vitro, and the knocking down of *AtACR2* results in As(V) sensitivity and As accumulation in *A. thaliana* seedlings [27]. However, another study suggested that a knockout of *AtACR2* does not affect As redox status and As accumulation in *A. thaliana* [28]. *Pteris vittata* ACR2p (PvACR2), a homolog of AtACR2, also exhibits arsenate reductase activity in vitro [29], but the arsenate reduction function of PvACR2 has not been confirmed in *P. vittata* yet. A novel arsenate reductase ARQ1/HAC1(Arabidopsis thaliana arsenate reductase QTL1/High Arsenic Content 1) had been identified in *A. thaliana* [11,13], and its homolog was also identified in rice and other plant species [30,31]. The HAC1 mainly functions to reduce As(V) to As(III) in the root, facilitating the efflux of As(III) back into the soil to limit both its accumulation in the root and transport to the shoot [11]. Recently, two HAC1 homologs, PvHAC1 and PvHAC2, were identified in *P. vittata*. The *PvHAC1* was mainly expressed in the rhizomes, while *PvHAC2* was predominantly expressed in the fronds of *P. vittata*. It is suggested that multiple arsenate reductases may work together in a tissue-specific manner and play a role in As-hyperaccumulation [32]. 

It is not yet known whether the two families of eukaryotic arsenate reductases, ACR2-related proteins and HAC1-related proteins, evolved from the same ancestor. The amino acid sequences of the ACR2 arsenate reductases and HAC1 arsenate reductases show less than 20% identity, but proteins from both families contain a rhodanese-like domain. Further, the HAC arsenate reductases have a consensus active site CX_4_R, whereas ACR2 arsenate reductases contain the HCX_5_R active site (Figure 1). It would be interesting to solve the structural differences between ACR2 and HAC1 to further understand the As(V) reduction mechanism mediated by these two proteins. 

Since eukaryotic arsenate reductases are not related to the prokaryotic ones, they may have evolved independently. An overall phylogeny analysis of bacterial and archaeal arsenate reductases suggested a single, early origin of the ArsC gene [33]. A recent study revealed a potential vertical and horizontal transfer history of these genes [34]. In contrast, it was suggested that the two prokaryotic arsenate reductases and yeast ACR2p underwent independent, convergent evolution based on crystallographic studies [35]. The *S. aureus* plasmid pI258 ArsC and the yeast ACR2p enzymes are homologs of phosphotyrosine phosphatases. HAC1 and ACR2p contain rhodanese-like domains of thiosulfate sulfur transferases [36]. Interestingly, the substrates of all three types of these enzymes (arsenate reductases, phosphatases, and sulfur transferases) are oxyanions (arsenate, phosphate, and thiosulfate, respectively) [10]. This evolutionary relatedness suggests that their common ancestor had an oxyanion binding site [19].

## 3. Arsenic Uptake and Extrusion Mechanisms, from Prokaryote to Eukaryote

As(III) and As(V) enter the cell via aquaporins and phosphate and hexose transporters, respectively [37,38]. Since arsenate is quickly reduced to As(III) by arsenate reductases within the cell, its major form there is As(III). The bulk of As(III) is then extruded out of the cells in a short time. For example, As(III) efflux typically accounts for 60% to 90% of the As(V) uptake by roots of rice and other plants [39]. 

An extrusion of As out of the cells is a major mechanism for As resistance in prokaryotic cells [17]. During the primordial ages of the earth, As(III) was the predominant As form. Bacteria first evolved an ArsRB operon, which contains two genes, *ArsR* and *ArsB*. The ArsR protein is a member of the SmtB/ArsR family of metalloregulatory proteins [40]. ArsR binds the promoter region of Ars operons, acting as a trans-acting transcriptional repressor. As(III) interacts with ArsR, which dissociates it from the DNA, thus allowing transcription of the operon [7]. The *arsB* encodes a membrane protein extruding As(III) out of the cytoplasm, thus diminishing As(III) accumulation [18]. After oxygen appeared in the atmosphere, arsenate became more abundant than As(III). Thus, ArsC was evolved to reduce As(V) to As(III) for extrusion by the ArsB efflux pump [7]. In addition, Sato and Kobayashi [41] reported the presence of the As(III) efflux pump in *Bacillus subtilis*, with homology to ACR3, a protein encoded by the yeast *S. cerevisiae*, which also confers As resistance [12]. Later it was found that almost every prokaryotic species have either an *ArsB* gene or an *ACR3* gene [18]. The *ArsB* genes exist mainly in Firmicutes and Gamma proteobacteria, but *ACR3* genes are predominantly found in Actinobacteria and Alpha proteobacteria [42]. Further resistance to higher levels of As and tight regulation of As resistance requires an additional two genes, *ArsA* and *ArsD* [22]. ArsA, an ATPase encoded by *ArsA*, could bind to ArsB and convert the As(III) carrier protein into a primary ATP-driven As(III) pump [43]. The ArsA–ArsB complex results in ATP-coupled As(III) extrusion independent of the electrochemical proton gradient, while ArsB needs electrochemical energy to efflux As(III) out of cells [18,43]. ArsD functions as an As(III) metallochaperone, which can bind As(III) and transfer it to the arsA [18]. Cells expressing the *ArsRDABC* operon are more resistant to As(V) and As(III) than those expressing only the *ArsRBC* genes [43]. It has been suggested that there is possible coevolution of ArsA with ArsB or Acr3 [44]. In *E. coli*, a glycerol facilitator, GlpF, which was first identified as an antimonite (SbIII) channel, is the major uptake pathway for As(III) [45]. In microbes, the As efflux is mainly conducted by As efflux transporter ArsB and ACR3 [8]. However, in some bacteria, for example, a legume symbiont *Sinorhizobium meliloti*, ArsB is replaced by another protein, aqpS, which belongs to the major intrinsic protein of the aquaporin superfamily, and functions as an efflux channel for As(III) [46]. Since aqpS is a channel, it can only mediate the downhill transport of As(III), whereas ArsB can transport As(III) against the electrochemical gradient for As. Thus, arsenic detoxification mediated by aqpS-ArsC occurs under As(V) conditions [30].

In most cases, ArsB is sufficient for bacterial arsenical resistance. In eukaryotic cells, the As extrusion mechanism is more complicated. No eukaryotic ArsB orthologs have been identified yet. In yeast, As(III) is extruded out of the cell via ScACR3 transporter [47]. Although the size of ScACR3 (404 residues, 45.8 kDa) and ArsB (429 residues, 45.6 kDa) is similar, and both of them catalyze extrusion of As(III) from the cytosol to the extracellular medium, they evolve independently, possess different structure, and have different substrate specificity [22]. ArsB confers resistance to arsenite and antimonite, whereas ScACR3 is an arsenite-specific resistance protein [12]. Although both of them operate as As/H^+^ antiporters, ArsB has 12 membrane-spanning helixes [48], whereas ScACR3 contains ten transmembrane domains [47]. In some lower plants, such as an As hyperaccumulator fern *P. vittata*, ACR3 homologs exist but have different functions [49]. PvACR3 is located at the tonoplast of *P. vittata* and mediates the detoxification of As by compartment As(III) in the vacuole. *PvACR3* is highly expressed in both sporophyte roots and gametophytes. A knockdown of *PvACR3* in the gametophyte results in an As(III)-sensitive phenotype [49]. However, when overexpressed in *A. thaliana*, PvACR3 was located at the plasma membrane and mediated As(III) efflux from the roots and translocation to the shoot [50,51]. Unlike PvACR3, PvACR3;1 localized to the vacuolar membrane conferred As sequestration when expressed in *A. thaliana* roots [52,53]. Recently, two more *ACR3* genes (*PvACR3;2* and *PvACR3;3*) were identified in *P. vittata*, which were mainly expressed in fronds of *P. vittata* [54]. PvACR3;2 was localized on the plasma membrane and mediated both AsIII efflux and translocation, while PvACR3;3 was localized to the vacuolar membrane, mediating As(III) sequestration into the vacuoles [54]. The duplication of *ACR3* genes suggests that they are critical for the As tolerance and hyperaccumulation in *P. vittata*. Until now, the ACR3 protein family was only identified in bacteria, fungi and non-angiosperm plants [49]. According to the phylogenetic analysis, the absence of *ACR3* genes in angiosperms and their presence in non-angiosperms, such as *Picea sitchensis* and *Welwitschia mirabilis*, suggests that ACR3 was lost in the angiosperms shortly after diverging from the gymnosperms [49]. The loss of *ACR3* in higher plants also suggests that they may use different transporters for As efflux. 

**Figure 2:**
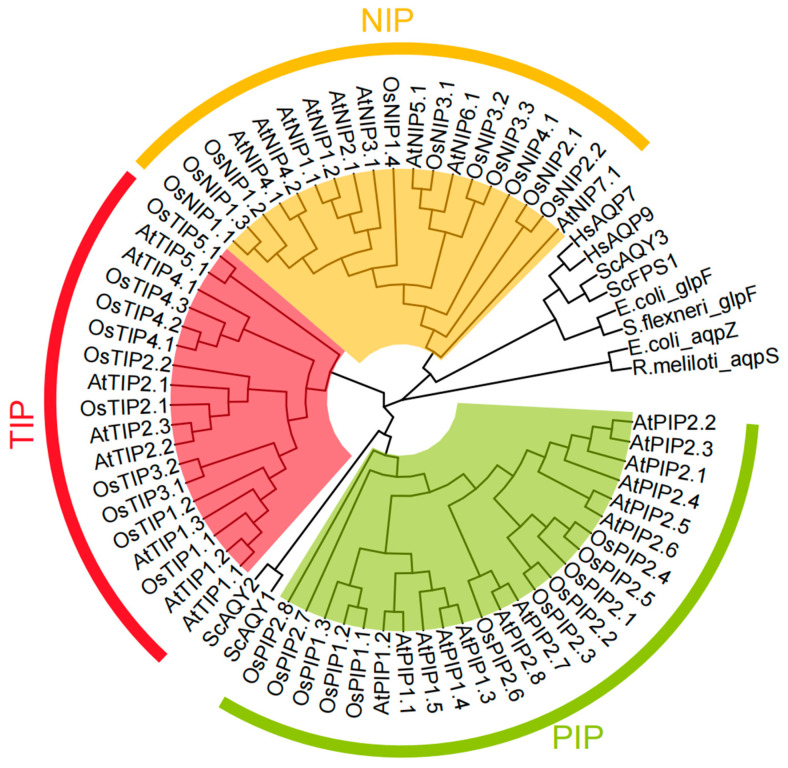
The phylogenetic tree of aquaporins from *E. coli*, *S. flexneri*, *R. meliloti*, S. *cerevisiae*, *A. thaliana*, *O. sativa*, and *H. sapiens*. Alignment of the amino acid sequences of aquaporins was made by Clustal W software using default parameters. The phylogenetic tree was made using MEGA X software by the Maximum Likelihood method, with 1000 Bootstrap replicates. The tree was finalized using the R software ggtree package. Different colors of arcs indicate different subfamilies. The accession numbers of the proteins are shown in Supplementary Table S2.

Aquaporins play critical roles in As uptake as well as extrusion, especially in eukaryotic cells. Although aquaporins are present in almost all living organisms, the number of aquaporins varies among organisms [55] (Figure 2). *E. coli* and yeast contain only two and four members of aquaporins, and the human genome has 11 members, while plants developed a larger aquaporin family [56,57]. For instance, *A. thaliana* and rice contain 35 and 34 members of aquaporins, respectively [56]. The *S. cerevisiae* FPS1, an ortholog of *E. coli* glycerol channel GlpF, mediates the uptake and efflux of glycerol from yeast cells [45,46]. The fact that a knockout of FPS1 enhances As(III) tolerance and that an expression of the constitutively open Fps1p channel causes hypersensitivity to As(III), suggested that Fps1p plays an important role in As(III) uptake [58]. The *S. cerevisiae* genome contains four aquaporin genes, *FPS1*, *AQY1*, *AQY2*, and *AQY3* [59]. FPS1 and AQY3 are similar, and both exhibit glycerol permeability. AQY1 and AQY2 are water channels. Since it is a bidirectional permease, FPS1 is also involved in As(III) efflux, which is critical for maintaining As(V) tolerance [60]. 

The functions of aquaporin proteins for As transport have been extensively studied in plant species. Based on their protein similarity and subcellular localization, aquaporins are divided into five major subgroups in higher plants, including nodulin 26-like intrinsic membrane proteins (NIPs), plasma membrane intrinsic proteins (PIPs), tonoplast intrinsic proteins (TIPs), small basic intrinsic proteins (SIPs), and uncategorized intrinsic proteins (XIPs) [61]. Multiple members of these subfamilies have been shown to play an important role in As(III) transport, with the NIPs being the most important. 

In *A. thaliana*, AtNIP1;1, AtNIP1;2, AtNIP3;1, AtNIP5;1, AtNIP6;1, and AtNIP7;1 have been demonstrated as As(III) transporters [62,63,64,65,66], although in different operational modes, such as As(III) uptake and translocation from root to shoot and from the xylem to the phloem. AtNIP1;1, AtNIP2;1, AtNIP3;1, AtNIP5;1, and AtNIP7;1 are mainly involved in As(III) uptake. AtNIP1;1 is localized at the plasma membrane of root epidermal cells and mediates As(III) uptake. The *atnip1;1* mutant exhibited 30% lower As accumulation than wild-type plants [63]. AtNIP5;1 also exhibited As(III) uptake transport activity in yeast expressing *AtNIP5;1* [66]. The predominant expression of *AtNIP5;1* in root epidermal, cortical, and endodermal cells suggested that it might also be involved in As(III) uptake [67,68]. The loss-of-function mutant of *AtNIP7;1* shows increased tolerance to As(III) and As(V), reduces the total As concentrations in plants, which indicates that AtNIP7;1 functions as an As(III) uptake transporter [62]. AtNIP1;2 and AtNIP3;1 were also suggested to play a role in As(III) uptake since both of them are expressed at the root epidermal tissues [65,69]. The translocation of As from root to shoot is one of the major factors affecting and determining the accumulation of this toxic metal in shoots and seeds. Arsenic translocation from root to shoot is mainly mediated by AtNIPs localized at vascular tissue in *A. thaliana*. AtNIP3;1 also plays an important role in the xylem loading of As(III) since it is also expressed in root pericycle cells [65]. As(III) is transported into seeds through the remobilization pathway of the phloem transport system [70,71]. Phloem transport is thought to account for 90% of the As(III) uploaded into rice grains [70]. AtNIP6;1 is predominantly expressed in nodal regions of shoots, especially the phloem region of vascular tissues [72]. The knockout mutants of *AtNIP6;1* also showed reduced As content in the seeds [73], which suggested that AtNIP6;1 might be involved in As(III) translocation from the xylem to the phloem. Since most of these NIP proteins have multiple substrates, including Si, B, and As(III) [64], it would be valuable to understand mechanisms of substrate specificity for the future. 

In rice, As(III) uptake is mainly mediated by OsNIP2;1, also known as a rice low-silicon transporter (OsLsi1), which is localized in a polar manner at the distal side of the plasma membrane of the exodermis and endodermis cells [74]. Beside, As(III) uptake, OsNIP2;1 is also involved in the uptake of various methylated As species, including monomethylarsonic acid (MMA) and dimethylarsinic acid (DMA) [75,76]. Other rice aquaporins proteins, including OsNIP1;1, OsNIP2;2, OsNIP3;1, OsNIP3;2, OsNIP3;3, OsPIP2;4, OsPIP2;6, and OsPIP2;7, also possess permeability to As(III) when expressed in yeast cells or *Xenopus laevis* oocytes [66,75,77,78]. However, their functions in As(III) accumulation in plants has not been fully elucidated yet. 

In addition, aquaporins in other plant species have also been reported to play a role in As(III) uptake and translocation. It has been found that PvTIP4;1 aquaporin in *P. vittata*, which encodes a plasma membrane-localized protein, is mainly expressed in the roots and mediates As(III) transport [79]. The heterologous expression of *PvTIP4;1* in *A. thaliana* enhances As accumulation and induces As sensitivity [79]. 

Arsenic efflux is also a major arsenic resistance mechanism in plants. Until now, only a few studies dealt with As(III) efflux transporters in *A. thaliana* [8]. Since neither ArsB nor ACR3 homologs were found in the *A. thaliana* genome, other transporter families, including aquaglyceroporins, might be involved in this process. This issue needs to be addressed in further studies. In rice, certain aquaporins such as OsNIP2;1, OsPIP2;4, OsPIP2;6, and OsPIP2;7 have been found to be able to mediate As(III) efflux when the cellular As(III) concentration is higher than outside since they mediate the As(III) transport across the membrane according to the concentration gradient [55]. Compared to wheat and barley, rice roots could more efficiently extrude a large amount of As(III) to either the rhizosphere or xylem [39,80]. When rice plants are treated with As(V), the amounts of As(III) extruded out account for 60–90% of the total As(V) uptake [39]. This process is partially mediated by OsNIP2;1, which accounts for about 15–20% of the total efflux of As(III) [39]. However, major As efflux transporters in rice have not been identified yet. 

## 4. Sequestration of As(III) Conjugated with Thiol Compounds in Yeast and Plants

Plants and fungi contain a specialized subcellular organelle, vacuole, to store nutrients, metabolites, as well as xenobiotics. Besides extruding As(III) out of the cells, yeast and plants develop their own As tolerance mechanism using the vacuolar compartmentation of As(III). In yeast, As(III) could be chelated with glutathione to form As(GS)_3_, which is then sequestered into the vacuole by a C-type ABC transporter, ScYCF1 (yeast cadmium factor 1), a homolog of the human MRP. ScYCF1 was first reported as a vacuolar glutathione *S*-conjugate pump that conferred cadmium resistance in *S. cerevisiae* [81]. Ghosh et al. [12] found that the disruption of ScYCF1 also increases the sensitivity to both As(III) and As(V). Vacuolar membrane vesicles from wild-type yeast expressing ScYCF1 can transport ^73^As(III) in the presence of MgATP and glutathione, while those of *Δycf1* do not have ^73^As(GS)_3_ transport activity. 

In plants, As(III) is also detoxified by being compartmentalized into the vacuoles, followed by complexing with thiol compounds, such as PCs (phytochelatins) [82,83]. PCs are glutathione-derived compounds synthesized via several key enzymes, including γ-glutamylcysteine synthetase and PC synthase. Arsenic may strongly induce PC synthesis [84,85]. Genes or enzymes involved in glutathione synthesis, metabolism, and transport are upregulated in rice seedlings upon exposure to As(V). The *A. thaliana* phytochelatin synthase mutant (*cad1*) is 10–20 times more sensitive to As(V) than the wild-type [86]. Overexpression of the bacterial γ-glutamylcysteine synthetase gene (*γ-ECS*) [87,88] or the *A. thaliana* PC synthase gene (*AtPCS1*) enhances As(V) tolerance [89,90]. The As(III)-PCs complexes are further sequestered into the vacuoles of plant cells for detoxification in a process mediated by ABC transporters. AtABCC1 and AtABCC2 contribute to the vacuolar sequestration of As(III)-PCs conjugates and As resistance in *A. thaliana* [14]. A double knockout mutant of *AtABCC1* and *AtABCC2* exhibits severe growth retardation upon As treatment. Rice OsABCC1 plays an important role in As resistance as well as As accumulation in grain [91]. OsABCC1, located at the vacuolar membrane of nodal phloem companion cells, compartmentalizes As and prevents its translocation to grains. Another C-type ABC transporter, OsABCC7, plays a role in the root-to-shoot translocation of As(III) [92]. *OsABCC7* is highly expressed in the roots and encodes a plasma membrane-localized protein. OsABCC7 is preferentially localized in the xylem parenchyma cells in the stele region of the primary and lateral roots, with the transport activity for both As(III)-phytochelatin and As(III)-glutathione complexes. The knockout mutants of *OsABCC7* showed significantly reduced As concentrations in xylem sap and shoots [92]. These results suggest that the vacuolar sequestration of As is a crucial mechanism for alleviating As toxicity and regulating As mobilization to grains in plants.

ScYCF1, HsMRP1, and plant ABCC1 proteins can detoxify arsenic by transporting As(GS)_3_ or As(III)-PCs complexes out of the cytosol. Both ScYCF1 and HsMRP1 transport As(GS)_3_, but plant ABCC1 proteins transport As(III)-PCs. The different substrate preferences of these proteins suggest that they may have some divergence during evolution. It would be worth studying what sequential or structural differences determine this substrate recognition. 

## 5. Other as Resistance Mechanisms Present in Both Prokaryotes and Eukaryotes

*Pseudomonas aeruginosa* strain DK2 has a specific pathway for As resistance, involving two novel genes conferring resistance to As(V), a glyceraldehyde 3-phosphate dehydrogenase (GAPDH) and an MFS transporter, ArsJ. Under this scenario, As(V) could be transformed to As(V) phosphoglycerate derivative, 1-arseno-3-phosphoglycerate (1As3PGA) by GAPDH, followed by its extrusion through ArsJ permease [16]. 

A similar system has been found in *P. vittata*, which consists of three essential genes that are significantly induced by As(V), namely glyceraldehyde 3-phosphate dehydrogenase (PvGAPC1), organic cation transporter 4 (PvOCT4), and glutathione S-transferase (PvGSTF1) [15]. PvGAPC1 has a higher affinity to As(V) than to phosphate, and converts As(V) into 1-arseno-3-phosphoglycerate (1As3PGA), which is transported into As-metabolizing vesicles by PvOCT4. In the vesicles, 1As3PGA further hydrolyzes, releasing As(V), which is reduced to As(III) by PvGSTF1. The As(III) may be further released into the central vacuole by membrane fusion with the vesicles. Although the subtle details of the operation of this pathway for As resistance are slightly different between *Pseudomonas aeruginosa* strain DK2 and *P. vittata*, it is highly possible that this As resistance pathway in the *P. vittata* evolved from *Pseudomonas aeruginosa* strain DK2. The systematic analysis of the genome sequences would be helpful for proving it. It should be noted, however, that this mechanism has not been found in angiosperms. 

## 6. Outstanding Questions to Be Solved in Future Research

Despite the significant efforts that have been made to reveal the mechanistic basis of arsenic resistance in different organisms, there is still a long way to go for a comprehensive understanding of this process, let alone the application of this knowledge for reducing arsenic accumulation in crops. In our view, future studies need to be focused on the following issues:

(1) How did the arsenic resistance trait evolve from prokaryotes to higher plants? For example, the arsenate reductases belong to different protein families in different organisms, such as ArsC protein in prokaryotes, ACR2 in yeast and fern, and HAC1 in higher plants. Although the ACR2 protein exists in higher plants, no clear evidence demonstrates its function as an arsenate reductase. It is interesting to know whether these proteins come from the same ancestor or have evolved separately.
(2) Are there any new mechanisms for arsenic resistance in higher plants? In prokaryotes, a rare case of mechanism for the detoxification of arsenate by converting arsenate to As(V) phosphoglycerate and transporting it out of the cell was reported [16]. A similar strategy has been identified in the As hyperaccumulating fern *P. vittata* [15]. However, such a mechanism has not been identified in yeast or higher plants. Does this pathway only exist in prokaryotes and *P. vittata*? In addition, the present review focuses on the tolerance mechanisms for inorganic arsenic species, while organic arsenic species are also present in the soil and should be dealt with by living organisms.
(3) We know that arsenate reductase, aquaglyceroporins, and ABC transporters participate directly in the transport of arsenic or arsenic-containing complexes. However, their regulation is still understood poorly, especially in higher plants. In bacteria arsRDABC operons, ArsR protein acts as a trans-acting transcriptional repressor that regulates the transcription of the operon [40]. Yeasts possess more complex regulation mechanisms for arsenic resistance. The AP-1-like protein Yap8p specifically contributes to arsenic resistance by mediating the arsenic-induced expression of *ACR2* and *ACR3* [93,94]. The stabilization of Yap8p was found to be regulated by the ubiquitin-proteasome pathway, which is mediated by the ubiquitin-conjugating enzyme Ubc4p [95]. The phosphorylation of As(III) uptake-transporter FPS1 by high-osmolarity glycerol kinase (HOG1) results in further ubiquitination and degradation in the vacuole, thus reducing arsenic uptake in yeast cells [96]. The most well-characterized member of the yeast AP-1 family, Yap1, is involved in the arsenic adaptation process through the regulation of the expression of the vacuolar pump encoded by YCF1 [93]. However, little is known about the regulatory mechanisms for the major components of arsenic resistance, such as ABCC1, aquaporins, PCS, and HAC1 in plants.

## 7. Conclusions

**Figure 3:**
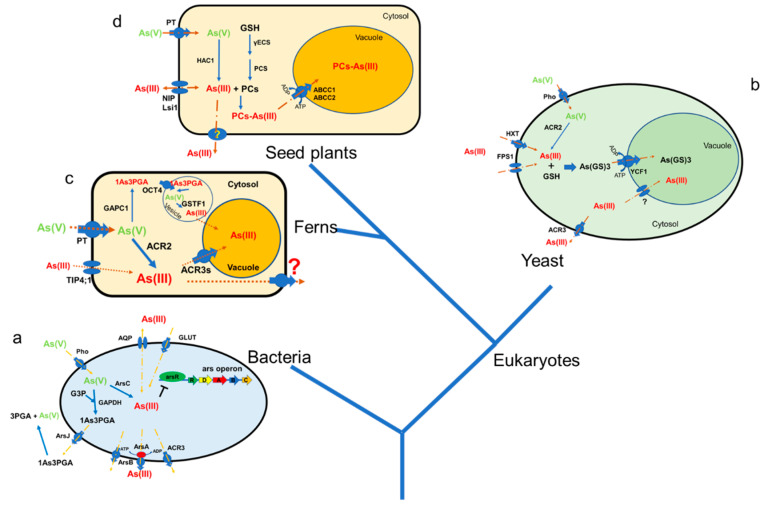
A simplified evolution scheme of arsenic resistance mechanisms mediated by As transporters in different organisms. (**a**). Arsenic resistance mechanisms in bacteria. As(V) enters bacteria cells via the phosphate uptake transporter (Pho) and is then reduced to As(III) by arsenate reductase ArsC. The bacteria cell detoxifies As(III) mainly by extruding it out by ArsB or ACR3 proteins. In some particular bacteria, As(V) can be transformed into 1-arseno-3-phosphoglycerate (1As3PGA) via glyceraldehyde 3-phosphate dehydrogenase (GAPDH). The 1As3PGA is then extruded by the ArsJ protein. (**b**) Arsenic tolerance mechanisms mediating As efflux and vacuolar sequestration in *Saccharomyces cerevisiae*. Arsenic enters yeast cells via FPS1 (fdp1 suppressor), HXTs (hexose transporter), and PHOs (phosphate transporter). Arsenate is reduced to As(III) by As(V) reductase ACR2, and cytosolic As(III) is extruded via ACR3 transporter or sequestrated into the vacuole by ScYCF1 after conjugation with glutathione. (**c**) Arsenic tolerance mechanisms in *P. vittata*. As(V), taken up by the phosphate transporter is reduced to As(III) by PvACR2, which is then translocated from the root to the frond. In both root and frond tissues, As(III) could be detoxified by sequestrating in the vacuole mediated by the PvACR3 transporter. Further, a bacterial-like mechanism for As(V) tolerance was evolved in *P. vittata*, which involves three genes, PvGAPC1, PvOCT4, and PvGSTF1. (**d**) Arsenic tolerance mechanisms in higher plants. Here, phosphate transporters and aquaporins mediate the uptake of As(V) and As(III), respectively. Inside the plant cell, As(V) is reduced to As(III) via the HAC1 protein. As(III) is then detoxified by sequestration into vacuole followed by conjugation with phytochelatins (PCs) or by extruding out of the cell via aquaporins or some unknown transporter. The As resistance mechanisms in higher plants gradually evolved from prokaryotes.

Foods contaminated with arsenic can potentially threaten human health. Reducing As content in the edible parts of crops needs a comprehensive understanding of arsenic resistance mechanisms, which include arsenate reduction, arsenic efflux, and arsenic sequestration. In this review, we summarized key As resistance mechanisms in various living systems, including prokaryotes, yeast, an As hyperaccumulator *P. vittata*, and higher plants (Figure 3), as well as compared the major differences in these mechanisms among different organisms. These As resistance traits have gradually evolved from prokaryotes to higher plants (Figure 3). Understanding the evolution of the arsenic resistance mechanisms from prokaryotic to higher plants may help us find new mechanisms and new genes conferring arsenic resistance in higher plants, which would be important for reducing As accumulation in edible crops.

## Data Availability

Not applicable.

## Supplementary Materials

The following supporting information can be downloaded at: www.mdpi.com/xxx/s1,Table S1: The accession number of the proteins used to generate phylogenetic tree of Figure 1; Table S2: The accession number of the proteins used to generate phylogenetic tree of Figure 2.

## References

[1] Singh R., Singh S., Parihar P., Singh V.P., Prasad S.M. (2015). Arsenic contamination, consequences and remediation techniques: A review. Ecotoxicol. Environ. Saf..

[2] Argos M., Kalra T., Rathouz P.J., Chen Y., Pierce B., Parvez F., Islam T., Ahmed A., Rakibuz-Zaman M., Hasan R. (2010). Arsenic exposure from drinking water, and all-cause and chronic-disease mortalities in Bangladesh (HEALS): A prospective cohort study. Lancet.

[3] Basu A., Saha D., Saha R., Ghosh T., Saha B. (2014). A review on sources, toxicity and remediation technologies for removing arsenic from drinking water. Res. Chem. Intermed..

[4] Hettick B.E., Canas-Carrell J.E., French A.D., Klein D.M. (2015). Arsenic: A Review of the Element’s Toxicity, Plant Interactions, and Potential Methods of Remediation. J. Agric. Food Chem..

[5] Zhu Y.G., Yoshinaga M., Zhao F.J., Rosen B.P. (2014). Earth Abides Arsenic Biotransformations. Annu. Rev. Earth Planet. Sci..

[6] Zhao F.J., McGrath S.P., Meharg A.A. (2010). Arsenic as a Food Chain Contaminant: Mechanisms of Plant Uptake and Metabolism and Mitigation Strategies. Annu. Rev. Plant Biol..

[7] Ben Fekih I., Zhang C.K., Li Y.P., Zhao Y., Alwathnani H.A., Saquib Q., Rensing C., Cervantes C. (2018). Distribution of Arsenic Resistance Genes in Prokaryotes. Front. Microbiol..

[8] Garbinski L.D., Rosen B.P., Chen J. (2019). Pathways of arsenic uptake and efflux. Environ. Int..

[9] Chen S.C., Sun G.X., Yan Y., Konstantinidis K.T., Zhang S.Y., Deng Y., Li X.M., Cui H.L., Musat F., Popp D. (2020). The Great Oxidation Event expanded the genetic repertoire of arsenic metabolism and cycling. Proc. Natl. Acad. Sci. USA.

[10] Mukhopadhyay R., Rosen B.P. (2002). Arsenate reductases in prokaryotes and eukaryotes. Environ. Health Perspect..

[11] Chao D.Y., Chen Y., Chen J.G., Shi S.L., Chen Z.R., Wang C.C., Danku J.M., Zhao F.J., Salt D.E. (2014). Genome-wide Association Mapping Identifies a New Arsenate Reductase Enzyme Critical for Limiting Arsenic Accumulation in Plants. PLoS Biol..

[12] Ghosh M., Shen J., Rosen B.P. (1999). Pathways of As(III) detoxification in Saccharomyces cerevisiae. Proc. Natl. Acad. Sci. USA.

[13] Sanchez-Bermejo E., Castrillo G., del Llano B., Navarro C., Zarco-Fernandez S., Martinez-Herrera D.J., del Puerto Y.L., Munoz R., Camara C., Paz-Ares J. (2014). Natural variation in arsenate tolerance identifies an arsenate reductase in Arabidopsis thaliana. Nat. Commun..

[14] Song W.Y., Park J., Mendoza-Cozatl D.G., Suter-Grotemeyer M., Shim D., Hortensteiner S., Geisler M., Weder B., Rea P.A., Rentsch D. (2010). Arsenic tolerance in Arabidopsis is mediated by two ABCC-type phytochelatin transporters. Proc. Natl. Acad. Sci. USA.

[15] Cai C., Lanman N.A., Withers K.A., DeLeon A.M., Wu Q., Gribskov M., Salt D.E., Banks J.A. (2019). Three Genes Define a Bacterial-Like Arsenic Tolerance Mechanism in the Arsenic Hyperaccumulating Fern Pteris vittata. Curr. Biol..

[16] Chen J., Yoshinaga M., Garbinski L.D., Rosen B.P. (2016). Synergistic interaction of glyceraldehydes-3-phosphate dehydrogenase and ArsJ, a novel organoarsenical efflux permease, confers arsenate resistance. Mol. Microbiol..

[17] Bhattacharjee H., Rosen B.P., Nies D.H., Silver S. (2007). Arsenic Metabolism in Prokaryotic and Eukaryotic Microbes. Molecular Microbiology of Heavy Metals.

[18] Yang H.C., Fu H.L., Lin Y.F., Rosen B.P. (2012). Pathways of Arsenic Uptake and Efflux. Curr. Top. Membr..

[19] Messens J., Silver S. (2006). Arsenate reduction: Thiol cascade chemistry with convergent evolution. J. Mol. Biol..

[20] Saltikov C.W., Newman D.K. (2003). Genetic identification of a respiratory arsenate reductase. Proc. Natl. Acad. Sci. USA.

[21] Ji G., Garber E.A., Armes L.G., Chen C.M., Fuchs J.A., Silver S. (1994). Arsenate reductase of Staphylococcus aureus plasmid pI258. Biochemistry.

[22] Rosen B.P. (1999). Families of arsenic transporters. Trends Microbiol..

[23] Bennett M.S., Guan Z., Laurberg M., Su X.D. (2001). Bacillus subtilis arsenate reductase is structurally and functionally similar to low molecular weight protein tyrosine phosphatases. Proc. Natl. Acad. Sci. USA.

[24] Bobrowicz P., Wysocki R., Owsianik G., Goffeau A., Ulaszewski S. (1997). Isolation of three contiguous genes, ACR1, ACR2 and ACR3, involved in resistance to arsenic compounds in the yeast Saccharomyces cerevisiae. Yeast.

[25] Mukhopadhyay R., Rosen B.P. (1998). Saccharomyces cerevisiae ACR2 gene encodes an arsenate reductase. FEMS Microbiol. Lett..

[26] Fauman E.B., Cogswell J.P., Lovejoy B., Rocque W.J., Holmes W., Montana V.G., Piwnica-Worms H., Rink M.J., Saper M.A. (1998). Crystal structure of the catalytic domain of the human cell cycle control phosphatase, Cdc25A. Cell.

[27] Dhankher O.P., Rosen B.P., McKinney E.C., Meagher R.B. (2006). Hyperaccumulation of arsenic in the shoots of Arabidopsis silenced for arsenate reductase (ACR2). Proc. Natl. Acad. Sci. USA.

[28] Liu W., Schat H., Bliek M., Chen Y., McGrath S.P., George G., Salt D.E., Zhao F.-J. (2012). Knocking Out ACR2 Does Not Affect Arsenic Redox Status in Arabidopsis thaliana: Implications for As Detoxification and Accumulation in Plants. PLoS ONE.

[29] Ellis D.R., Gumaelius L., Indriolo E., Pickering I.J., Banks J.A., Salt D.E. (2006). A novel arsenate reductase from the arsenic hyperaccumulating fern Pteris vittata. Plant Physiol..

[30] Shi S.L., Wang T., Chen Z., Tang Z., Wu Z.C., Salt D.E., Chao D.Y., Zhao F.J. (2016). OsHAC1;1 and OsHAC1;2 Function as Arsenate Reductases and Regulate Arsenic Accumulation. Plant Physiol..

[31] Xu J.M., Shi S.L., Wang L., Tang Z., Lv T.T., Zhu X.L., Ding X.M., Wang Y.F., Zhao F.J., Wu Z.C. (2017). OsHAC4 is critical for arsenate tolerance and regulates arsenic accumulation in rice. New Phytol..

[32] Li X.Y., Sun D., Feng H.Y., Chen J.X., Chen Y.S., Li H.B., Cao Y., Ma L.N.Q. (2020). Efficient arsenate reduction in As-hyperaccumulator Pteris vittata are mediated by novel arsenate reductases PvHAC1 and PvHAC2. J. Hazard. Mater..

[33] Jackson C.R., Dugas S.L. (2003). Phylogenetic analysis of bacterial and archaeal arsC gene sequences suggests an ancient, common origin for arsenate reductase. BMC Evol. Biol..

[34] Dunivin T.K., Yeh S.Y., Shade A. (2019). A global survey of arsenic-related genes in soil microbiomes. BMC Biol..

[35] Mukhopadhyay R., Rosen B.P., Pung L.T., Silver S. (2002). Microbial arsenic: From geocycles to genes and enzymes. FEMS Microbiol. Rev..

[36] Hofmann K., Bucher P., Kajava A.V. (1998). A model of Cdc25 phosphatase catalytic domain and CDK-interaction surface based on the presence of a rhodanese homology domain. J. Mol. Biol..

[37] Meharg A.A., Macnair M.R. (1990). An altered phosphate uptake system in arsenate-tolerant *Holcus lanatus* L.. New Phytol..

[38] Clemens S., Ma J.F. (2016). Toxic Heavy Metal and Metalloid Accumulation in Crop Plants and Foods. Annu. Rev. Plant Biol..

[39] Zhao F.J., Ago Y., Mitani N., Li R.Y., Su Y.H., Yamaji N., McGrath S.P., Ma J.F. (2010). The role of the rice aquaporin Lsi1 in arsenite efflux from roots. New Phytol..

[40] Busenlehner L.S., Pennella M.A., Giedroc D.P. (2003). The SmtB/ArsR family of metalloregulatory transcriptional repressors: Structural insights into prokaryotic metal resistance. FEMS Microbiol. Rev..

[41] Sato T., Kobayashi Y. (1998). The ars operon in the skin element of Bacillus subtilis confers resistance to arsenate and arsenite. J. Bacteriol..

[42] Achour A.R., Bauda P., Billard P. (2007). Diversity of arsenite transporter genes from arsenic-resistant soil bacteria. Res. Microbiol..

[43] Dey S., Rosen B.P. (1995). Dual-Mode of Energy Coupling by the Oxyanion-Translocating Arsb Protein. J. Bacteriol..

[44] Castillo R., Saier M.H. (2010). Functional Promiscuity of Homologues of the Bacterial ArsA ATPases. Int. J. Microbiol..

[45] Luyten K., Albertyn J., Skibbe W.F., Prior B.A., Ramos J., Thevelein J.M., Hohmann S. (1995). Fps1, a yeast member of the MIP family of channel proteins, is a facilitator for glycerol uptake and efflux and is inactive under osmotic stress. EMBO J..

[46] Sutherland F.C., Lages F., Lucas C., Luyten K., Albertyn J., Hohmann S., Prior B.A., Kilian S.G. (1997). Characteristics of Fps1-dependent and -independent glycerol transport in Saccharomyces cerevisiae. J. Bacteriol..

[47] Wysocki R., Bobrowicz P., Ulaszewski S. (1997). The Saccharomyces cerevisiae ACR3 gene encodes a putative membrane protein involved in arsenite transport. J. Biol. Chem..

[48] Wu J., Tisa L.S., Rosen B.P. (1992). Membrane topology of the ArsB protein, the membrane subunit of an anion-translocating ATPase. J. Biol. Chem..

[49] Indriolo E., Na G., Ellis D., Salt D.E., Banks J.A. (2010). A Vacuolar Arsenite Transporter Necessary for Arsenic Tolerance in the Arsenic Hyperaccumulating Fern Pteris vittata Is Missing in Flowering Plants. Plant Cell.

[50] Chen Y., Xu W., Shen H., Yan H., Xu W., He Z., Ma M. (2013). Engineering arsenic tolerance and hyperaccumulation in plants for phytoremediation by a PvACR3 transgenic approach. Environ. Sci. Technol..

[51] Wang C., Na G., Bermejo E.S., Chen Y., Banks J.A., Salt D.E., Zhao F.J. (2018). Dissecting the components controlling root-to-shoot arsenic translocation in Arabidopsis thaliana. New Phytol..

[52] Chen Y.S., Hua C.Y., Chen J.X., Rathinasabapathi B., Cao Y., Ma L.Q. (2019). Expressing Arsenite Antiporter PvACR3;1 in Rice (*Oryza sativa* L.) Decreases Inorganic Arsenic Content in Rice Grains. Environ. Sci. Technol..

[53] Chen Y.S., Hua C.Y., Jia M.R., Fu J.W., Liu X., Han Y.H., Liu Y.G., Rathinasabapathi B., Cao Y., Ma L.Q. (2017). Heterologous Expression of Pteris vittata Arsenite Antiporter PvACR3;1 Reduces Arsenic Accumulation in Plant Shoots. Environ. Sci. Technol..

[54] Chen J.X., Cao Y., Yan X., Chen Y., Ma L.Q. (2021). Novel PvACR3;2 and PvACR3;3 genes from arsenic-hyperaccumulator Pteris vittata and their roles in manipulating plant arsenic accumulation. J. Hazard. Mater..

[55] Deng F.L., Liu X., Chen Y.S., Rathinasabapathi B., Rensing C., Chen J., Bi J., Xian P., Ma L.N.Q. (2020). Aquaporins mediated arsenite transport in plants: Molecular mechanisms and applications in crop improvement. Crit. Rev. Environ. Sci. Technol..

[56] Deshmukh R.K., Sonah H., Belanger R.R. (2016). Plant Aquaporins: Genome-Wide Identification, Transcriptomics, Proteomics, and Advanced Analytical Tools. Front. Plant Sci..

[57] Madeira A., Moura T.F., Soveral G. (2016). Detecting Aquaporin Function and Regulation. Front. Chem..

[58] Wysocki R., Chery C.C., Wawrzycka D., Van Hulle M., Cornelis R., Thevelein J.M., Tamas M.J. (2001). The glycerol channel Fps1p mediates the uptake of arsenite and antimonite in Saccharomyces cerevisiae. Mol. Microbiol..

[59] Sabir F., Loureiro-Dias M.C., Soveral G., Prista C. (2017). Functional relevance of water and glycerol channels in Saccharomyces cerevisiae. FEMS Microbiol. Lett..

[60] Maciaszczyk-Dziubinska E., Migdal I., Migocka M., Bocer T., Wysocki R. (2010). The yeast aquaglyceroporin Fps1p is a bidirectional arsenite channel. FEBS Lett..

[61] Maurel C., Boursiac Y., Luu D.T., Santoni V., Shahzad Z., Verdoucq L. (2015). Aquaporins in Plants. Physiol. Rev..

[62] Isayenkov S.V., Maathuis F.J.M. (2008). The Arabidopsis thaliana aquaglyceroporin AtNIP7;1 is a pathway for arsenite uptake. FEBS Lett..

[63] Kamiya T., Tanaka M., Mitani N., Ma J.F., Maeshima M., Fujiwara T. (2009). NIP1;1, an Aquaporin Homolog, Determines the Arsenite Sensitivity of Arabidopsis thaliana. J. Biol. Chem..

[64] Mitani-Ueno N., Yamaji N., Zhao F.J., Ma J.F. (2011). The aromatic/arginine selectivity filter of NIP aquaporins plays a critical role in substrate selectivity for silicon, boron, and arsenic. J. Exp. Bot..

[65] Xu W.Z., Dai W.T., Yan H.L., Li S., Shen H.L., Chen Y.S., Xu H., Sun Y.Y., He Z.Y., Ma M. (2015). Arabidopsis NIP3;1 Plays an Important Role in Arsenic Uptake and Root-to-Shoot Translocation under Arsenite Stress Conditions. Mol. Plant.

[66] Bienert G.P., Thorsen M., Schussler M.D., Nilsson H.R., Wagner A., Tamas M.J., Jahn T.P. (2008). A subgroup of plant aquaporins facilitate the bi-directional diffusion of As(OH)3 and Sb(OH)3 across membranes. BMC Biol..

[67] Tanaka M., Fujiwara T. (2021). Three regions of the NIP5;1 promoter are required for expression in different cell types in Arabidopsis thaliana root. Plant Signal. Behav..

[68] Takano J., Wada M., Ludewig U., Schaaf G., von Wiren N., Fujiwara T. (2006). The Arabidopsis major intrinsic protein NIP5;1 is essential for efficient boron uptake and plant development under boron limitation. Plant Cell.

[69] Wang Y., Li R., Li D., Jia X., Zhou D., Li J., Lyi S.M., Hou S., Huang Y., Kochian L.V. (2017). NIP1;2 is a plasma membrane-localized transporter mediating aluminum uptake, translocation, and tolerance in Arabidopsis. Proc. Natl. Acad. Sci. USA.

[70] Carey A.M., Scheckel K.G., Lombi E., Newville M., Choi Y., Norton G.J., Charnock J.M., Feldmann J., Price A.H., Meharg A.A. (2010). Grain unloading of arsenic species in rice. Plant Physiol..

[71] Li N., Wang J., Song W.Y. (2016). Arsenic Uptake and Translocation in Plants. Plant Cell Physiol..

[72] Tanaka M., Wallace I.S., Takano J., Roberts D.M., Fujiwara T. (2008). NIP6;1 is a boric acid channel for preferential transport of boron to growing shoot tissues in Arabidopsis. Plant Cell.

[73] Lindsay E.R., Maathuis F.J.M. (2017). New Molecular Mechanisms to Reduce Arsenic in Crops. Trends Plant Sci..

[74] Ma J.F., Tamai K., Yamaji N., Mitani N., Konishi S., Katsuhara M., Ishiguro M., Murata Y., Yano M. (2006). A silicon transporter in rice. Nature.

[75] Ma J.F., Yamaji N., Mitani N., Xu X.Y., Su Y.H., McGrath S.P., Zhao F.J. (2008). Transporters of arsenite in rice and their role in arsenic accumulation in rice grain. Proc. Natl. Acad. Sci. USA.

[76] Li R.Y., Ago Y., Liu W.J., Mitani N., Feldmann J., McGrath S.P., Ma J.F., Zhao F.J. (2009). The rice aquaporin Lsi1 mediates uptake of methylated arsenic species. Plant Physiol..

[77] Sun S.K., Chen Y., Che J., Konishi N., Tang Z., Miller A.J., Ma J.F., Zhao F.J. (2018). Decreasing arsenic accumulation in rice by overexpressing OsNIP1;1 and OsNIP3;3 through disrupting arsenite radial transport in roots. New Phytol..

[78] Chen Y., Sun S.K., Tang Z., Liu G., Moore K.L., Maathuis F.J.M., Miller A.J., McGrath S.P., Zhao F.J. (2017). The Nodulin 26-like intrinsic membrane protein OsNIP3;2 is involved in arsenite uptake by lateral roots in rice. J. Exp. Bot..

[79] He Z.Y., Yan H.L., Chen Y.S., Shen H.L., Xu W.X., Zhang H.Y., Shi L., Zhu Y.G., Ma M. (2016). An aquaporin PvTIP4;1 from Pteris vittata may mediate arsenite uptake. New Phytol..

[80] Su Y.H., McGrath S.P., Zhao F.J. (2010). Rice is more efficient in arsenite uptake and translocation than wheat and barley. Plant Soil.

[81] Li Z.S., Lu Y.P., Zhen R.G., Szczypka M., Thiele D.J., Rea P.A. (1997). A new pathway for vacuolar cadmium sequestration in Saccharomyces cerevisiae: YCF1-catalyzed transport of bis(glutathionato)cadmium. Proc. Natl. Acad. Sci. USA.

[82] Pickering I.J., Prince R.C., George M.J., Smith R.D., George G.N., Salt D.E. (2000). Reduction and coordination of arsenic in Indian mustard. Plant Physiol..

[83] Raab A., Schat H., Meharg A.A., Feldmann J. (2005). Uptake, translocation and transformation of arsenate and arsenite in sunflower (Helianthus annuus): Formation of arsenic-phytochelatin complexes during exposure to high arsenic concentrations. New Phytol..

[84] Schmoger M.E.V., Oven M., Grill E. (2000). Detoxification of arsenic by phytochelatins in plants. Plant Physiol..

[85] Sneller F.E.C., Van Heerwaarden L.M., Kraaijeveld-Smit F.J.L., Ten Bookum W.M., Koevoets P.L.M., Schat H., Verkleij J.A.C. (1999). Toxicity of arsenate in Silene vulgaris, accumulation and degradation of arsenate-induced phytochelatins. New Phytol..

[86] Ha S.B., Smith A.P., Howden R., Dietrich W.M., Bugg S., O’Connell M.J., Goldsbrough P.B., Cobbett C.S. (1999). Phytochelatin synthase genes from arabidopsis and the yeast Schizosaccharomyces pombe. Plant Cell.

[87] Li Y.J., Dankher O.P., Carreira L., Smith A.P., Meagher R.B. (2006). The shoot-specific expression of gamma-glutamylcysteine synthetase directs the long-distance transport of thiol-peptides to roots conferring tolerance to mercury and arsenic. Plant Physiol..

[88] Dhankher O.P., Li Y.J., Rosen B.P., Shi J., Salt D., Senecoff J.F., Sashti N.A., Meagher R.B. (2002). Engineering tolerance and hyperaccumulation of arsenic in plants by combining arsenate reductase and gamma-glutamylcysteine synthetase expression. Nat. Biotechnol..

[89] Gasic K., Korban S.S. (2007). Transgenic Indian mustard (*Brassica juncea*) plants expressing an Arabidopsis phytochelatin synthase (AtPCS1) exhibit enhanced As and Cd tolerance. Plant Mol. Biol..

[90] Li Y.J., Dhankher O.P., Carreira L., Lee D., Chen A., Schroeder J.I., Balish R.S., Meagher R.B. (2004). Overexpression of phytochelatin synthase in Arabidopsis leads to enhanced arsenic tolerance and cadmium hypersensitivity. Plant Cell Physiol..

[91] Song W.Y., Yamaki T., Yamaji N., Ko D., Jung K.H., Fujii-Kashino M., An G., Martinoia E., Lee Y., Ma J.F. (2014). A rice ABC transporter, OsABCC1, reduces arsenic accumulation in the grain. Proc. Natl. Acad. Sci. USA.

[92] Tang Z., Chen Y., Miller A.J., Zhao F.J. (2019). The C-type ATP-Binding Cassette Transporter OsABCC7 Is Involved in the Root-to-Shoot Translocation of Arsenic in Rice. Plant Cell Physiol..

[93] Wysocki R., Fortier P.K., Maciaszczyk E., Thorsen M., Leduc A., Odhagen A., Owsianik G., Ulaszewski S., Ramotar D., Tamas M.J. (2004). Transcriptional activation of metalloid tolerance genes in Saccharomyces cerevisiae requires the AP-1-like proteins Yap1p and Yap8p. Mol. Biol. Cell.

[94] Kumar N.V., Yang J.B., Pillai J.K., Rawat S., Solano C., Kumar A., Grotli M., Stemmler T.L., Rosen B.P., Tamas M.J. (2016). Arsenic Directly Binds to and Activates the Yeast AP-1-Like Transcription Factor Yap8. Mol. Cell Biol..

[95] Di Y.J., Tamas M.J. (2007). Regulation of the arsenic-responsive transcription factor Yap8p involves the ubiquitin-proteasome pathway. J. Cell Sci..

[96] Mollapour M., Piper P.W. (2007). Hog1 mitogen-activated protein kinase phosphorylation targets the yeast Fps1 aquaglyceroporin for endocytosis, thereby rendering cells resistant to acetic acid. Mol. Cell Biol..

